# Tear Film Steroid Profiling in Dry Eye Disease by Liquid Chromatography Tandem Mass Spectrometry

**DOI:** 10.3390/ijms18071349

**Published:** 2017-06-24

**Authors:** Damiana Pieragostino, Luca Agnifili, Ilaria Cicalini, Roberta Calienno, Mirco Zucchelli, Leonardo Mastropasqua, Paolo Sacchetta, Piero Del Boccio, Claudia Rossi

**Affiliations:** 1 Department of Medical Oral and Biotechnological Sciences, University “G. d’Annunzio” of Chieti-Pescara, 66100 Chieti, Italy; mirco.zucchelli@unich.it (M.Z.); ps@unich.it (P.S.); claudia.rossi@unich.it (C.R.); 2 Analytical Biochemistry and Proteomics Laboratory, Research Centre on Aging and Translational Medicine (Ce.S.I-MeT), University “G. d’Annunzio” of Chieti-Pescara, 66100 Chieti, Italy; ilaria.cicalini@unich.it (I.C.); p.delboccio@unich.it (P.D.B.); 3 Opthalmic Clinic, Department of Medicine and Aging Science, “G. d’Annunzio” University of Chieti-Pescara, 66100 Chieti, Italy; l.agnifili@unich.it (L.A.); roberta.calienno@gmail.com (R.C.); leonardo.mastropasqua@unich.it (L.M.); 4 Department of Pharmacy, University “G. d’Annunzio” of Chieti-Pescara, 66100 Chieti, Italy

**Keywords:** dry eye disease, tears, steroids, mass spectrometry, biomarkers, LC-MS/MS

## Abstract

Dry eye disease (DED) is a multifactorial disorder of the ocular surface unit resulting in eye discomfort, visual disturbance, and ocular surface damage; the risk of DED increases with age in both sexes, while its incidence is higher among females caused by an overall hormonal imbalance. The role of androgens has recently investigated and these hormones were considered to have a protective function on the ocular surface. In order to correlate DED to tear steroid levels, a robust, specific, and selective method for the simultaneous quantification of cortisol (CORT), corticosterone (CCONE), 11-deoxycortisol (11-DECOL), 4-androstene-3,17-dione (ADIONE), testosterone (TESTO), 17α-hydroxyprogesterone (17-OHP), and progesterone (PROG) was developed and applied for the analysis of tear samples. The method involves a simple extraction procedure of steroids from tears collected on Schirmer strips, followed by a high-performance liquid chromatography-tandem mass spectrometry (HPLC-MS/MS) analysis. In total, tear samples from 14 DED female patients and 13 healthy female controls were analysed and, CORT, ADIONE, and 17-OHP response levels resulted significantly decreased in dry eye patients respect to controls. The receiver operating characteristic (ROC) curve obtained by the combination of these three steroids (AUC = 0.964) demonstrated the good diagnostic power of the differential tear steroids in identifying DED. In conclusion, the present method made it possible, for the first time, to study steroid profiling directly in tear fluid.

## 1. Introduction

The ocular surface is an integrated unit comprising corneal and conjunctival epithelia, meibomian glands (MGs), main and accessory lachrymal glands, and trigeminal neurons; their dysfunction results in a scarce or unstable tear film that causes dry eye, with a higher incidence among postmenopausal women [1].

Dry eye disease (DED), as a multifactorial disease, presents a complex aetiology and pathophysiology [2]. It is widely known that systemic and metabolic dysfunctions, such as lower intake of omega-3 and omega-6 fatty acids, menopause, acne and ovarian dysfunction, diabetes mellitus, and use of systemic medications (antihistamines, b-blockers, antidiuretics, and antidepressants) could favour the development of DED and increase the severity of the disease [3]. Sex hormone imbalance plays a crucial role in the pathophysiology of different ocular surface diseases including dry eye, with a different impact of oestrogens and steroids. In detail, their imbalance may significantly increase the risk and modify the course of DED, since serum oestrogen levels are strongly associated with the development and progression of dry eye [4]. This is also supported by the fact that women are more likely to experience DED during periods of substantial hormonal alteration, such as pregnancy, lactation, oral contraceptive use and after the menopause [5,6].

Experimental and human studies have demonstrated that androgen levels are essential for the normal lacrimal gland function and for structural organisation, and that prolactin and oestrogens play important roles in providing an adequate hormonal environment for optimal tear production [7]. In fact, it was demonstrated that systemic replacement treatment with combined esterified oestrogen and methyltestosterone may be efficacious in treating dry eye syndrome of various aetiologies [8]. Moreover, receptors for androgens, oestrogens, progesterone and prolactin have been identified in several ocular tissues, including the main lacrimal gland and MGs [9], leading to the hypothesis that tear steroids may have a key role in the physiology of these glands.

To date, the relationship between sex hormone levels and tear production remains complex, and it is unclear how sex hormones regulate the functional activity of ocular glandular tissues.

Since tears have already revealed important insights for studying eye disorders [10,11,12], and considering our experience in the development of liquid chromatography-tandem mass spectrometry (LC-MS/MS) methods for the determination of metabolites related to pathologies [13,14,15,16], we aimed to measure steroid levels directly on tear samples, for the first time. Thus, a robust, specific, and selective method was developedfor the simultaneous quantification of the following steroids: cortisol (CORT); corticosterone (CCONE); 11-deoxycortisol (11-DECOL); 4-androstene-3,17-dione (ADIONE); testosterone (TESTO); 17α-hydroxyprogesterone (17-OHP); and progesterone (PROG). The proposal methodology was applied for the analysis of a small casuistry of tear samples from 14 female dry eye patients and 13 female controls.

## 2. Results

### 2.1. Sensitivity, Linearity, Imprecision, and Recovery

**Figure 1:**
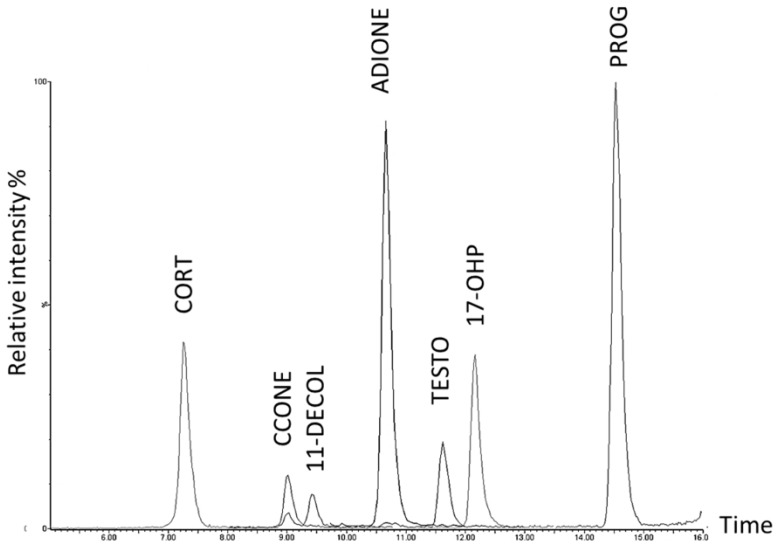
The chromatogram obtained at the upper concentration for each analyte through the proposed high-performance liquid chromatography-tandem mass spectrometry (HPLC-MS/MS) method shows a good resolution among the investigated steroids. Additionally, the two isomers corticosterone (CCONE) and 11-deoxycortisol (11-DECOL) are chromatographically resolved.

Following the criteria described above limit of detection (LOD) and lower limit of quantification (LLOQ) were established to be: 0.30 and 0.50 ng/mL for CORT; 0.1 and 0.29 ng/mL for CCONE; 0.05 and 0.10 ng/mL for 11-DECOL; 0.05 and 0.08 ng/mL for ADIONE; 0.02 and 0.08 ng/mL for TESTO; 0.05 and 0.08 ng/mL for 17-OHP. The only exception was PROG, for which LOD and LLOQ coincided with the lowest concentration Schirmer strip calibrator (0.05 ng/mL). The correlation between steroid concentration and signal intensities, including two blank Schirmer strip calibrators, was linear: from 0.50 to 20.80 ng/mL for CORT; from 0.10 to 1.00 ng/mL for 11-DECOL; from 0.08 to 2.91 ng/mL for ADIONE; from 0.08 to 1.00 ng/mL for TESTO; from 0.08 to 4.20 ng/mL for 17-OHP; and from 0.05 to 4.20 ng/mL for PROG. For CCONE the linearity was observed from 0.29 to 4.03 ng/mL, but the method resulted to be not linear for this steroid. CCONE has the same nominal molecular mass as 11-DECOL, and even if the chromathographic conditions were able to chromatographically resolve CCONE and 11-DECOL (Figure 1), the signal for CCONE after LC-MS/MS analysis of Schirmer strip samples was found to be low, reflecting the typical low endogenous concentration of this steroid. Table 1 shows the investigated linear ranges, the calculated calibration functions, and the corresponding correlation coefficients (*r^2^*). The *r^2^* for all analytes was >0.993 over their concentration ranges, and all calculated concentrations for the calibrators were within ±10% of the assigned values. The intra- and inter-assay coefficient of variation (CV) and deviation from assigned values was ≤15.2% and ≤13.9% for all analytes, respectively (Table 2) [17]. The mean analytical recovery from the extraction at each Quality Control (QC) level for all steroids, without internal standard correction, was also summarized in Table 2. Recovery using analyte peak areas exclusive of internal standard normalization gives an indication of the efficiency of analyte extraction resulting from the chosen method of sample preparation. According to the principle of isotope dilution internal standardization, the addition of isotopically labelled internal standards for each analyte prior to the extraction should compensate for any variation in ionisation efficiency due to residual matrix effects and for losses in recovery of the extraction process [13].

### 2.2. Steroid Profiling of Dry Eye and Healthy Control Tear Samples by LC-MS/MS Analysis

The LC-MS/MS method we developed was employed for the analysis of tear samples collected on Schirmer paper strips in a total of 27 people divided in 14 DED female patients and 13 healthy control females. Since about the 25% of tears samples had steroid concentration below our defined LLOQ, with the exception for CORT and PROG, and considering the limited number of patients for this first application, we decided to express our data in term of response (analyte peak area/internal standard peak area) for all the seven steroids.

**Figure 2:**
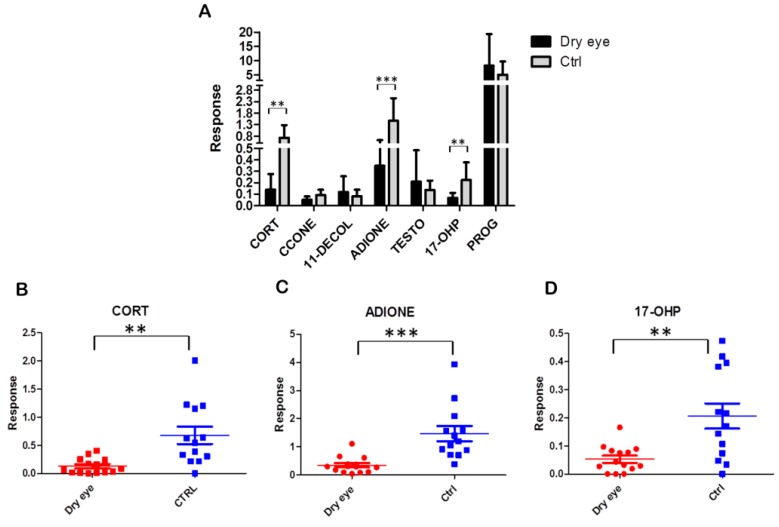
(**A**) Tear steroid profiling of dry eye (*n* = 14) and of healthy control patients (*n* = 13). Data are mean values and bars represent the corresponding standard deviations (SD); (**B**–**D**) Tear sample distribution for the significantly different steroids: CORT, ADIONE and 17-OHP response levels, respectively. ** indicates *p* < 0.01 and *** indicates *p* < 0.001.

**Figure 3:**
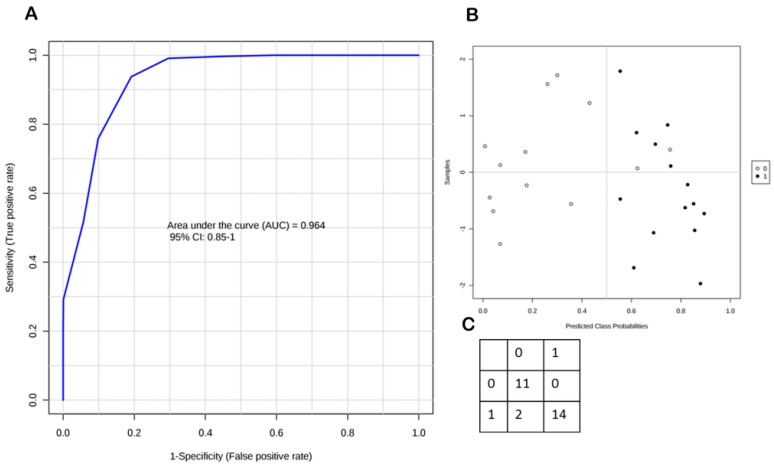
(**A**) Receiver operating characteristic (ROC) curve performed on the three significantly different steroids calculated (CORT, ADIONE, and 17-OHP) resulting in an AUC = 0.964; (**B**) The 100 cross-validation is shown, underlying good predictivity of the proposed model (*p* < 0.01) in differentiating dry eye patients from healthy people; (**C**) Samples are grouped in class 0 and 1 for controls and dry eye patients, respectively.

In order to define data distribution in each group, data matrix was statistically processed performing D’Agostino and Pearson omnibus normality test. Once normality was accepted, Student’s t-test was performed, otherwise the Mann Whitney test was carried out to assess the significantly expressed variables between the groups. Response levels were measured for all the seven steroids. CORT, ADIONE, and 17-OHP response levels resulted significantly decreased (*p*-value < 0.05) in dry eye patient samples compared to control samples. CCONE response level also resulted decreased in this comparison, although for this steroid we did not achieve the optimal conditions after the validation assay procedure. Figure 2 shows tear steroid profiling of dry eye and in healthy control patients (panel A). Data are mean values and bars represent the corresponding standard deviations (SD): ** indicates *p* < 0.01, and *** indicates *p* < 0.001. In Figure 2, panel B–D represent tear sample distribution for CORT, ADIONE and 17-OHP response level, respectively. In order to evaluate the diagnostic predictive power, we performed receiver operating characteristic (ROC) curve based on the three significantly different steroids obtained. After this investigation, the area under the curve (AUC) was 0.964, highlighting good sensitivity and specificity in discriminating the two study groups (Figure 3, panel A). We also performed the 100 cross-validation showing the predicted class probabilities of each sample, underlying good predictivity of the proposed model (*p* < 0.01) in discriminating dry eye patients (class 1) from healthy people (class 0) (Figure 3, panel B,C) [18].

## 3. Discussion

DED describes a group of tear film disorders that cause irritation and ocular surface damage. The most common subtypes of DED include aqueous and lipid deficiency, although most patients with DED have abnormalities in both tear components [19]. Considering dry eye prevalence and cost, known risk, and underlying pathogenesis of DED seems to be determinant to achieve effective prevention and treatments. Age (menopause) and female sex are considered as valid risk factors for DED development as stated in the literature [20]. In recent years, LC-MS/MS has had a pivotal role in routine clinical chemistry thanks to a wide range of applicability, easy sample preparation, and high analytical specificity, which allows for the identification, characterization and quantification of chemical compounds as target analytes based on their respective molecular masses and fragmentation patterns [21,22]. This technique allows the simultaneous analysis of a large number of metabolites from many different biological matrices in order to investigate multifactorial disease, as in the case of DED.

Even if different LC-MS/MS methods for the determination of a number of steroids from serum, plasma, dry-blood spot and urine samples has been already described, in this work for the first time we present a robust and sensitive LC-MS/MS method that allows us to analyse seven tear steroids by performing only one extraction procedure from tears collected on Schirmer strip [23,24,25,26]. We have demonstrated good sensitivity since we are able to detect up to 0.3 ng/mL for CORT, 0.05 ng/mL for 11-DECOL, ADIONE, 17-OHP and PROG, and 0.02 ng/mL for TESTO in tear samples. It can be an advantage since to date, the clinical used immunoassay for serum sex hormones have not been able to reliably measure the low concentrations, especially regarding TESTO in females and children [27]. It is also to consider that usually in clinical practice, it is needed one assay for each steroid of interest, while LC-MS/MS methods give the opportunity to monitor multiple analytes in a single analysis. Therefore, to test the seven steroids in tears by using the conventional immunoassay methods it would be necessary to perform seven different sample collection. The methodology described involves a simple extraction of target steroids (CORT, CCONE, 11-DECOL, ADIONE, TESTO, 17-OHP and PROG) from tears collected on Schirmer strip to obtain a specific profile through the multiple reaction monitoring (MRM) acquisition mode for each analyte using two transition (quantifier and qualifier ions) to ensure lack of interferences. Thus, our LC-MS/MS method proved to have good specificity, sensitivity, and also good linearity and intra- and inter-assay imprecision. The use of LC-MS/MS allowed us to obtain chromatographic resolution of the analytes of interest, even for CCONE and 11-DECOL, which have the same nominal molecular mass. Unfortunately linearity for CCONE was not achieved.

Many association of DED metabolic traits have been already reported, in particular it has been highlighted a relationship between DED and serum steroids. Vehof et al. found a strong significant association between DED and decreased levels of some serum androgens, suggesting the use of androgens as a potential pathway for the treatment of DED [28]. In fact, Bizzarro et al. proved that patients with DED and Sjögren syndrome showed a significant increase of tear volume in the Schirmer test and a decrease in the Bijsterveld score after oral testosterone undecanoate treatment with respect to placebo patients [29]. A correlation between DED and serum steroids has been observed, but it is unknown if it is possible to confirm the same relationship with the tear steroids. This feature is exactly the starting point of our pilot study.

Following a careful development and validation of an LC-MS/MS method for the measure of seven steroids in tears collected on paper strips, a small cohort of DED patients and controls was recruited and analysed for the determination of a tear steroid profiling potentially related to DED.

Statistical investigations of our data showed a significant reduction in tear response levels for CORT, ADIONE and 17-OHP in DED patients when compared to healthy controls. As also reported by Vehof et al. [28], a significant association was found for DED and decreased levels of some androgens. It is worth to mention that androgen levels should influence the structure and function of the lacrimal and Meibomian gland, with decreased androgen levels leading to lower tear volume, reduced tear film stability through decreased quality and quantity of meibomian gland lipids, decreased tear turnover rate, and hyperosmolarity [30,31]. ADIONE and 17-OHP are both secreted into the blood circulation by the adrenal glands [30]. Since ADIONE and 17-OHP tear levels resulted to be decreased in the case of DED, it is interesting to note that a positive correlation was demonstrated between these two steroids by Rudnicka et al. [32]. Moreover, CORT, a glucocorticoid, is produced by adrenal glands, in particular in the zona fasciculata of the adrenal cortex. Since CORT is one of the end product of steroidogenesis and derived by 17-OHP, we may speculate about a possible link between the decreased 17-OHP tear levels and the lower tear levels observed for CORT in DED patients. Interestingly, sex hormone PROG showed an opposite trend, even if not significant, increasing in dry eyes patients compared to controls. Higher levels of PROG is consistent with literature data that suggests a greater DED incidence during pregnancy, lactation, and oral contraceptive use [5,6].

In summary, this work revealed for the first time that tears represent a precious source of information for the study of the pathophysiologic state of the eye. In particular, in this pilot study steroid tear levels were measured and correlated to DED. Even if only a small cohort of tear samples were analysed, our innovative LC-MS/MS application added evidence of steroid level alteration in the tears of DED patients. Since the exact relationship between serum and tear steroid levels remains unknown, it could be interesting to study these two biological fluids from a large cohort of DED patients.

## 4. Materials and Methods

### 4.1. Patients

Dry eye patients and controls had to show a best correct visual acuity of 8/10, a mean intra-ocular pressure lower than 18 mm Hg, a central cornea thickness ranging from 530 to 570 μm, normal dilated funduscopy. At the moment of enrollment, patients with DED were not on therapy and did not receive topical steroids during the last 2 months. Exclusion criteria were diabetes mellitus, ocular lymphoma, sarcoidosis, autoimmune deficiency syndrome, corneal dystrophy, and non-DED-related ocular surface inflammatory diseases, systemic or topical therapy potential affecting the corneal status, glaucoma, topical therapy with steroids or nonsteroidal anti-inflammatory drugs, use of contact lenses, and previous ocular surgery. Exclusion criteria for normal controls were history of systemic or topical therapy, ocular or systemic diseases in the previous 12 months, pregnancy, and contact lens wear. Both of the eyes were evaluated, but one eye per subject was randomly chosen (using a computer-generated random number list) for statistical analysis.

### 4.2. Samples

All tear samples were collected at Opthalmic Clinic of University “G d’Annunzio” of Chieti-Pescara between September 2016 and February 2017. Tear samples from healthy and dry eye patients were collected on graduated strip for dry eye testing (Schirmer test I). Dry eye was diagnosed according to the International Dry Eye Workshop criteria [2]. Schirmer test I results were expressed as the length of the strip that was wet after 5 min. The Schirmer strips were purchased from EasyOpht (Busto Arsizio, VA, Italy). Once folded, the Schirmer paper strip at the mark, tears were collected asking the patients to look up and pulling the lower lid gently downward. After 5 min, the strip was removed from the eye and the length of the moistened area was measured using the millimeter scale on the strip. Then the filter paper was placed in a 2.0 mL Eppendorf tube, left dry at room temperature and stored at −80 °C. The Schirmer tests were considered as positive for dry eye diagnosis when strips were wet below 10 millimeters in five minutes. In case of the strip becomes completely wet before the indicated time, it may be removed in advance. This procedure was performed contemporarily for right and left eye for each patient. To preserve anonymity, full drug and medical histories were not available other than dry eye diagnosis and treatment. For this pilot study 13 healthy female and 14 dry eye female patients were analysed (from 25 to 73 years old), with three samples collected for each individual. In particular 10 patients presented an untreated dry eye without a diagnosis of autoimmune diseases, 2 patients were affected by dry eye thyroidism related and 1 patient affected by Sjogren disease (new diagnosis). More than 80% of patients with DED presented with a severity level 2, according to criteria previously reported [2]. All subjects gave their informed consent for inclusion before they participated in the study. The study was conducted in accordance with the Declaration of Helsinki.

### 4.3. Materials

CORT, CCONE, 11-DECOL, ADIONE, TESTO, 17-OHP and PROG were purchased from Sigma-Aldrich^®^ (St. Louis, MO, USA). ^2^H_3_-CORT, ^2^H_8_-CCONE, ^2^H_5_-11-DECOL, ^2^H_5_-ADIONE, ^2^H_5_-TESTO, ^2^H_8_-17-OHP, ^2^H_9_-PROG were from CHS^TM^ MSMS Steroids Kit, PerkinElmer^®^ (Turku, Finland). Water (H_2_O), methanol (MeOH), acetonitrile (ACN) LC-MS grade were from Romil-Pure chemistry^®^ (Cambridge, UK). Formic acid and ethanol LC-MS grade were from Sigma-Aldrich^®^ (St. Louis, MO, USA). HPLC solvent additive was from CHS MSMS Steroids Tool Box, PerkinElmer^®^ (Turku, Finland).

### 4.4. Preparation of Standard Solutions, Calibrators and Quality Controls

Each endogenous steroid (1 mg) was dissolved in ethanol (stock solution) and stored at −20 °C. Stock solutions of each compound were diluted in methanol/water 50:50 with a final concentration of 10 µg/mL (tuning solution) and stored at −20 °C. The internal standard mixture (IS mix) from PerkinElmer^®^ kit was reconstituted with 1.25 mL of ACN and stored at −20 °C. Before the extraction procedure, the Daily Precipitation Solution (DPS) containing Internal Standards was prepared by diluting 1:1000 the IS mix in two consecutive steps with ACN with 0.1% formic acid. After diluting the tuning solution, we obtained the working solutions for each steroid of interest as follow: CORT 50 ng/mL, CCONE, TESTO, 17-OHP and PROG 5 ng/mL, 11-DECOL and ADIONE 10 ng/mL. Calibrators were prepared from each working solution to achieve final concentrations as reported in Table 3. The same procedure was used to obtain QC samples that, considering the small concentration range of interest, resulted to coincide with L2, L4 and L6 calibrator levels (Table 3).

### 4.5. Sample Preparation

Calibrators and QCs in Schirmer paper strips were prepared wetting each paper strip with 90 µL of calibrator and QC solutions for each levels of calibration. Calibrators, QCs, tear samples collected on Schirmer strip were cut into 2–3 mm paper pieces and transferred into 2.0 mL microcentrifuge tube (Eppendorf^®^, Hamburg, Germany), paying attention to wash the required equipment with MeOH before each sample preparation. After adding 200 μL of DPS containing IS, each sample was gently mixed (20 °C, 15 min) in a Thermomixer (Eppendorf^®^) and then centrifuged (4210 rcf, 20 °C, 30 min). The organic layer (135 µL) was transferred into a new 1.5 mL tube and dried in a SpeedVac for 30 min. The residue was then reconstituted with 90 μL of H_2_O/MeOH 60:40, gently mixed in a Thermomixer (20 °C, 15 min), briefly centrifuged and finally transferred into polypropylene vial (Waters Corporation, Milford, MA, USA). The vials were capped, gently mixed, and placed in the system autosampler for analysis.

### 4.6. LC-MS/MS Analysis

The LC-MS/MS system was a HPLC Alliance HT 2795 Separations Module coupled to Quattro Ultima Pt ESI tandem quadrupole mass spectrometer (Waters Corporation, Milford, MA, USA). The system operated in positive electrospray ionization mode using MassLynx v4.1 software (Waters). For HPLC analysis, the Luna^®^ 3 μm C8, 100 Å, 50 × 3 mm column with Security Guard Cartridges, C8, 4.0 × 2.0 mm as guard column (Phenomenex^®^, USA) was used. Autosampler injections of 50 μL were made into sample loop in full mode. The mobile phase comprised a binary solvent system: 98% H_2_O and 2% MeOH (Solvent A) and 100% MeOH (Solvent B), both containing 0.025% of solvent additive. The initial solvent composition was 65% A and 35% B. The mobile phase gradient profile involved three steps: increasing from the initial conditions to 45% B within 4.0 min and then to 65% B within 9.0 min holding for 0.5 min before reaching 100% B until the end of the analysis. The total run time was 18.00 min, injection to injection. The flow rate was 0.35 mL/min and the column was maintained at 45 °C.

The mass spectrometer ionization source settings were optimized for maximum precursor ion yields for each steroid. This was achieved by injecting the tuning solution for each individual compound, all the parameters are summarized in Table 4. Two mass transitions were optimized for each analyte, with a single transition being used to monitor the corresponding deuterated internal standards. The first transition was used to quantify the target analyte and the second to qualify the identity of the target compound using a confirmatory ion ratio. The capillary voltage was 3.5 kV, source temperature was 120 °C, desolvation temperature was 400 °C, and the collision cell gas pressure was 3.5 × 10^−3^ mbar argon. The inter-channel and inter-scan delay times were 0.02 and 0.1 s, respectively. The dwell time was 0.45 s for CORT, 0.30 for CCONE and ADIONE, 0.25 for 11-DECOL, TESTO and 17-OHP, 0.35 for PROG. The same dwell time parameters as the ones of the endogenous compounds were used for the respective internal standards. Functions 1–7 (Table 4) refer to the MRM experiments created for each analyte.

### 4.7. Quantification

Data processing and quantification were performed using the QuanLynx 4.1 software (Waters Corporation, Milford, MA, USA) provided with the instrument. Calibration was performed through linear regression with reciprocal fit weighting to ensure maximum accuracy at the lower concentration range.

### 4.8. Assay Performance Characteristics

The LOD and the LLOQ were determined as the lowest concentration steroid spiked into Schirmer strip calibrators, giving a minimum signal-to-noise ratio (S/N) >3:1 for LOD and >8:1 for LLOQ in replicate analyses (*n* = 10). The method linearity was evaluated using Schirmer strip calibrators, including two blank Schirmer strip calibrators (with and without addition of internal standard solution) over a period of 5 days. Linearity was defined as deviation from the assigned calibrator values of ≤10%, with the exception of the LLOQ/lowest calibrator for which deviation of ≤15% was accepted. Precision and accuracy for each steroid were evaluated intra- and inter-day using QC material described above. For intra-assay imprecision, a sequence of five replicates of QC in Schirmer strips were analysed in a single batch, and for inter-assay studies, each QC was measured in singleton in batches over a 5-day period [13]. The intra- and inter-assay imprecision were expressed as a CV and deviation from assigned values. The analyte recovery indicates the efficiency of the extraction process and was evaluated following the strategy reported by Matuszewsky et al. [33]. Thus recovery was assessed spanning the analytical range of the assay by analysis of the Schirmer strip QCs and conducted for each steroid. Recovery was calculated as the analyte peak area of the extracted QC in Schirmer strip as a percentage of the peak area of a blank Schirmer strip spiked to the equivalent concentration after the extraction process [21]. Analyses were conducted in triplicate.

### 4.9. Statistical Analysis

Data obtained by LC-MS/MS analysis of tear samples on Schirmer strip from dry eye and control patients were statistically investigated using GraphPad Prism (GraphPad Software, Inc., La Jolla, CA, USA) and and MetaboAnalyst statistical analysis module [18].

## 5. Conclusions

In conclusion, we present for the first time a robust, specific, and selective LC-MS/MS method for the simultaneous quantification of seven steroids from tear samples. The method was, also, applied to a small casuistry of tear samples from 14 female DED patients and 13 female controls, showing a significant reduction in tear response levels for CORT, ADIONE, and 17-OHP in DED patients respect to healthy people. After statistical investigation, our proposal model demonstrated a good predictive power in identifying DED patients. Our data confirm the idea that tears can be used as a precious source of information for the study pathophysiologic state of the eye and of other different disorders.

## Figures and Tables

**Table 1:** Concentration ranges, calculated calibration functions, and correlation coefficients (*r^2^*). ^a^ b1 and b0 are mean values (*n* = 5). ^b^ Weight = 1/*x^2^*. Cortisol (CORT); 4-androstene-3,17-dione (ADIONE); testosterone (TESTO); 17α-hydroxyprogesterone (17-OHP); and progesterone (PROG).

Analyte	Concentration Range (ng/mL)	Calibration Function (*y* = b1(±SD)*x* + b0(±SD)) ^a,b^	*R^2^*
CORT	0.3–20.08	*y* = 0.396(±0.04)*x* + 0.026(±0.160)	0.996
11-DECOL	0.05–1.00	*y* = 3.041(±0.942)*x* + 0.077(±0.052)	0.994
ADIONE	0.05–2.91	*y* = 9.492(±0.584)*x* + 0.204(±0.054)	0.994
TESTO	0.02–1.00	*y* = 9.276 (±2.446)*x* + 0.171(±0.103)	0.993
17-OHP	0.05–4.20	*y* = 4.372 (±1.218)*x* − 0.053(±0.045)	0.993
PROG	0.05–4.20	*y* = 11.015 (±1.789)*x* + 0.279(±0.182)	0.994

**Table 2:** Intra- and inter-day precision and accuracy. Intra- and inter-assay precision, expressed as coefficient of variation (%CV), and recovery data evaluated at each Quality Control (QC) level. Standard deviation (SD).

Intra-Assay					Inter-Assay			Recovery %	
Compound	QC Level	Mean Response	SD	%CV	Mean Response	SD	%CV	Mean	SD
CORT	QC1	0.13	0.02	13.47	0.14	0.01	8.43	50.12	13.18
	QC2	0.52	0.01	2.35	0.52	0.03	5.12	33.12	0.73
	QC3	2.88	0.15	5.09	2.57	0.13	5.14	44.29	1.98
11-DECOL	QC1	0.27	0.02	7.97	0.15	0.02	12.41	46.99	6.64
	QC2	0.67	0.01	1.63	0.72	0.06	7.83	67.45	6.67
	QC3	2.19	0.11	4.98	2.26	0.21	9.48	58.18	2.83
ADIONE	QC1	0.83	0.04	4.99	0.99	0.03	3.15	23.26	7.16
	QC2	2.14	0.10	4.77	2.30	0.15	6.72	22.01	2.26
	QC3	11.99	0.13	1.12	11.85	0.32	2.73	24.53	2.23
TESTO	QC1	0.67	0.09	13.82	0.56	0.05	8.77	19.50	2.92
	QC2	2.14	0.31	14.67	1.92	0.09	4.51	37.46	0.48
	QC3	6.39	0.73	11.49	5.29	0.74	13.94	44.85	5.91
17-OHP	QC1	0.21	0.03	15.24	0.35	0.04	10.84	22.78	9.92
	QC2	0.78	0.07	8.86	1.04	0.06	5.99	30.61	6.49
	QC3	6.25	0.17	2.77	6.76	0.62	9.15	50.64	2.60
PROG	QC1	0.83	0.03	3.20	1.01	0.07	6.90	39.56	4.31
	QC2	2.26	0.21	9.54	2.95	0.19	6.70	30.60	1.92
	QC3	13.92	0.10	0.72	14.77	1.22	8.30	40.43	7.50

**Table 3:** Concentration levels (ng/mL) for calibrators and QC materials of each steroid monitored in the LC-MS/MS method of analysis are summarized.

Analyte	Calibration Levels (ng/mL)							QC Levels (ng/mL)		
	L1	L2	L3	L4	L5	L6	L7	QC1	QC2	QC3
CORT	0.30	0.50	1.00	1.51	3.61	8.66	20.80	0.50	1.51	8.66
CCONE	0.05	0.10	0.20	0.29	0.70	1.68	4.03	0.10	0.29	1.68
11-DECOL	0.05	0.07	0.10	0.20	0.40	0.70	1.00	0.07	0.20	0.70
ADIONE	0.05	0.08	0.10	0.20	0.49	1.17	2.91	0.08	0.20	1.17
TESTO	0.02	0.05	0.08	0.15	0.30	0.60	1.00	0.05	0.15	0.60
17-OHP	0.05	0.08	0.12	0.29	0.70	1.69	4.20	0.08	0.29	1.69
PROG	0.05	0.08	0.12	0.29	0.70	1.69	4.20	0.08	0.29	1.69

**Table 4:** MS/MS operating conditions. Multiple reaction monitoring (MRM) functions and settings for detection of steroids are shown. Italics denotes qualifier ion.

MRM Function	Time Window (min)	Analyte	Transitions (*m*/*z*)	Cone Volts	Coll Energy (eV)
1	5.0–8.5	CORT	363.2 > 120.8	100	20
		*CORT*	*363.2 > 96.9*	*100*	*20*
		^2^H_3_-CORT	366.2 > 120.8	100	20
2	8.0–10.5	CCONE	347.3 > 120.8	100	18
		*CCONE*	*347.3 > 96.8*	*100*	*20*
		^2^H_8_-CCONE	355.3 > 124.8	100	18
3	8.0–10.8	11-DECOL	347.3 > 108.8	100	22
		*11-DECOL*	*347.3 > 96.8*	*100*	*20*
		^2^H_5_-11-DECOL	352.3 > 112.8	100	22
4	9.7–12.0	ADIONE	287.2 > 96.9	100	18
		*ADIONE*	*287.2 > 108.8*	*100*	*20*
		^2^H_5_-ADIONE	292.2 > 99.9	100	18
5	10.5–13.0	TESTO	289.2 > 96.8	100	19
		*TESTO*	*289.2 > 108.8*	*100*	*18*
		^2^H_5_-TESTO	294.2 > 99.8	100	19
6	11.2–13.5	17-OHP	331.2 > 96.8	100	21
		*17-OHP*	*331.2 > 108.9*	*100*	*23*
		^2^H_8_-OHP	339.2 > 99.8	100	21
7	13.5–16.0	PROG	315.2 > 96.9	100	18
		*PROG*	*315.2 > 108.8*	*100*	*19*
		^2^H_9_-PROG	324.2 > 99.9	100	18
